# Combination Therapy of Carnosic Acid and Methotrexate Effectively Suppressed the Inflammatory Markers and Oxidative Stress in Experimental Arthritis

**DOI:** 10.3390/molecules27207115

**Published:** 2022-10-21

**Authors:** Martin Chrastina, Silvester Poništ, Jaroslav Tóth, Szilvia Czigle, Ľudmila Pašková, Veronika Vyletelová, Karol Švík, Katarína Bauerová

**Affiliations:** 1 Institute of Experimental Pharmacology and Toxicology, Centre of Experimental Medicine, Slovak Academy of Sciences, Dúbravská cesta 5826/9, SK-841 41 Bratislava, Slovakia; 2 Department of Pharmacology, Jessenius Faculty of Medicine in Martin, Comenius University Bratislava, Malá Hora 10701/4A, SK-036 01 Martin, Slovakia; 3 Department of Pharmacognosy and Botany, Faculty of Pharmacy, Comenius University Bratislava, Odbojárov 10, SK-832 32 Bratislava, Slovakia; 4 Department of Cell and Molecular Biology of Drugs, Faculty of Pharmacy, Comenius University Bratislava, Odbojárov 10, SK-832 32 Bratislava, Slovakia

**Keywords:** carnosic acid, methotrexate, adjuvant arthritis, combination therapy, inflammation, autoimmune disease, oxidative stress

## Abstract

Background: Combination therapy with methotrexate (MTX) is the most common therapeutic strategy used for the treatment of patients with rheumatoid arthritis (RA). In this study, we combined the natural compound carnosic acid (CA) with MTX to reduce inflammation and oxidative stress in adjuvant arthritis (AA). Methods: AA was induced in 6–8 rats per group. MTX was administrated twice a week at a dose of 0.3 mg/kg b.w., while CA was administered daily at a dose of 100 mg/kg both in monotherapy and in combination with MTX. Plasma samples were collected on the 14th, 21st, and 28th day. Body weight and hind paw volume were measured once a week. Results: We found that, mainly, the CA + MTX combination significantly reduced the hind paw swelling, the levels of IL-17A, MMP-9, and MCP-1 in plasma, and GGT activity in joint homogenates. The mRNA expression of HO-1, catalase, and IL-1β in the liver were significantly improved by CA + MTX only. Our results indicate that adding CA to MTX treatment could be a good therapeutic option for patients suffering from RA. Conclusions: The addition of CA to methotrexate treatment significantly improved its efficacy in decreasing the development of AA by inhibiting the markers of inflammation and oxidative stress.

## 1. Introduction

Rheumatoid arthritis (RA) is an autoimmune inflammatory disease that has approximately 0.5% occurrence in the population worldwide [1]. It is characterized by the production of many pro-inflammatory agents such as interleukins, matrix proteinases, oxygenases, and others [2]. As an inflammatory disease, RA is also characterized by the production of reactive oxygen species (ROS), which potentiate damage to tissue, cartilage, and bone and inflammation itself. In addition to patients’ health conditions, RA also has an economic and social impact on patients’ lives [3]. The causes of RA are still unknown, with few possible theories; therefore, there is need for research to elucidate these causes.

In recent years, the best therapeutic strategy has been proven to be an early diagnosis of RA, as an immediate beginning of therapeutic intervention improves clinical outcomes and reduces ongoing joint damage and disability [4]. Treatment options for RA include nonsteroidal anti-inflammatory drugs (NSAIDs), selective cyclooxygenase-2 (COX-2) inhibitors, corticosteroids, biologic disease-modifying antirheumatic drugs (bDMARDs), classic csDMARDs (methotrexate, hydroxychloroquine, etc.), and targeted synthetic DMARDs. Unfortunately, there is still no causal treatment for RA; only symptomatic approaches prevail in therapy. Methotrexate (MTX) has remained a fundament of RA treatment for decades [5]. However, its administration is limited due to toxic adverse effects such as mucous ulceration, cytopenia, nausea, liver damage, and cachexia [6]. MTX inhibits polyglutamate, aminoimidazole-4-carboxamide ribonucleotide (AICAR) transformylase, leading to intracellular accumulation of AICAR and increased adenosine release; adenosine binds to cell surface adenosine receptors (particularly A2A) and suppresses many inflammatory and immune reactions [7]. Patients receive an initial dose of 7.5 to 15 mg per week till reaching 20 to 30 mg per week [8]. However, many patients are intolerant or experience the persistence of moderate to severe disease activity despite csDMARD monotherapy or csDMARD combination therapy [9,10]. Despite there being many available therapeutic options for RA, the EULAR European Task Force has stressed that a large percentage of patients will not reach or maintain remission of clinical symptoms or low RA activity over time, or it will be necessary to stop treatment(s) due to tolerability or safety issues [11].

Adjuvant arthritis (AA) is an animal model of RA that is often used because of its shared features with RA. The similarities of both RA and AA include the manifestation of weight loss, swelling of acral body parts, production of inflammatory cytokines, and an activated immune system [12]. AA is used to determine the effectiveness of various experimental treatments in preclinical studies.

Cytokines that are expressed in the joints during the early stages of AA inflammation include IL-17, IFN-γ, and TNF-α, as well as cytokines implicated in macrophage stimulation. Increased levels of IL-4, IL-6, MCP-1, and TGF-β in systemic circulation can be observed as inflammation progresses in the joint. TNF-α, IL-1β, IL-21, and IL-17 all contribute to the pathology of this disorder [13]. Systemic serum/plasma increases in TNF-α, interleukin IL-17A, TGF-β, and chemokine MCP-1, together with local IL-1α/β and TGF-β enrichment, and local lymphoid hyperplasia precedes the onset of clinical disease and joint damage. Systemic up-regulation of IL-6, IL-17, TNF-α, TGF-β, IL-18, MCP-1, receptor activator of nuclear factor-kappa beta ligand (RANKL), and prostaglandin E2 during acute and chronic AA is present together with systemic leukocytosis and CD4 + T cell increase in the blood and spleen [14].

Metalloproteinases, also known as matrix metalloproteinases (MMPs), are enzymes that degrade proteins and require the presence of active metal atoms. There are more than 20 types of MMPs and they promote cell migration through proteolytic degradation of the extracellular basement. MMPs are up-regulated in cancers and in inflamed regions. MMPs are frequently found in the abnormal disease status of inflammatory responses, in inflammatory pulmonary lesions, arteriosclerotic smooth muscles, arthritis, and tumor metastases. Among two similar types of MMP, MMP-2 is known as gelatinase-A with 72 kDa, while MMP-9 is termed as gelatinase-B with 92 kDa. Both play a key role in this action [15].

Chemokines, including MCP-1/CCL2 and RANTES/CCL5, are highly expressed in the joints of patients with RA; they promote leukocyte migration into synovial tissue. RA is characterized by the infiltration of macrophages and lymphocytes in the joint, mediated in part by chemokines [16]. CC chemokines, MCP-1/CCL2 and RANTES/CCL5, are chemotactic for monocytes and T lymphocytes [17,18], and are detected in RA synovial fluid [19].

Gamma-glutamyl transferase is an important component of inflammatory processes since its activity is closely connected with the overall antioxidant status of the organism. The elevated expression and activity of GGT in joint tissue is a good marker for synovial inflammation, thus molecules able to reduce the activity of GGT could be promising in therapy.

Natural substances have been used for centuries to treat different diseases. A great number of experimental works performed with these natural substances in recent years have enabled a better understanding of molecular pathways and explained their mechanism of action. Combinations of standard therapy and natural substances are already used in some diagnoses, for example, in different types of cancer [20]. Active substances responsible for the biological effects were often discovered alongside the studies of natural extracts. These substances, responsible for bioactivities, can be used in experiments to potentiate the effect of standard treatment.

As the pharmacokinetic premise is that with a higher dosage of a drug there is a higher chance of the appearance of side effects, a lowered patient compliance is to be expected as well. Therefore, the prospect of a therapy that employs lower (even subtherapeutic) standard therapy doses in combination with a natural compound can lead to the maintenance of the same effect as that achieved by standard therapy in therapeutic doses.

Carnosic acid (CA) is a phenolic diterpene that was discovered in sage (*Salvia*), but rosemary (*Rosmarinus officinalis*) contains a higher concentration of CA; therefore, it is used as its primary source [21,22]. CA’s low stability in solutions is why CA changes into carnosol [23,24]. CA has demonstrated protective qualities in pathologies such as cancer, diabetes, and neurodegenerative disease. Its anti-inflammatory properties have been described and linked to the inhibition of MAPK, nuclear factor-kappa B (NF-κB), and the FoxO3a signaling pathway [10]. CA also has anxiolytic properties, which can improve life quality [25]. Rosemary has been described as efficient in reducing obesity and metabolic syndrome by inhibiting lipid absorption and decreasing lipid accumulation in the liver [26]. As higher reactive oxygen species production is present during inflammation, CA’s antioxidant properties can also lead to beneficial effects in RA and other inflammatory diseases [27]. CA was found to inhibit LPS-induced activation of mouse microglia, thus decreasing the release of inflammatory cytokines such as IL-1 and IL-6 [28]. CA has also been reported to decrease NO production associated with inducible NO synthase [29]. When CA was encapsulated in bovine serum albumin nanoparticles (CA-BSA-NPs), RT-PCR analysis showed an up-regulation of the GCLC gene and a down-regulation of the BCL-2 and COX-2 genes in cancer cells treated with CA-BSA-NPs compared to untreated cells. CA-BSA-NPs could be a promising drug form for treating colorectal and breast cancer [30]. Den Hartogh et al. [31] studied the effects of CA on palmitate-induced insulin-resistant L6 myotubes and 3T3L1 adipocytes. Palmitate-treated cells decreased the insulin-stimulated glucose uptake, GLUT4 transporter on the plasma membrane, and the activation of protein kinase B (Akt). CA reduced the deleterious effect of palmitate and renewed the insulin-stimulated glucose uptake, activation of Akt, and GLUT4 transporter levels.

This study aims to find the possible beneficial effect of CA in RA in monotherapy, as well as in combination therapy with methotrexate (MTX). The hypothesis is that combining the subtherapeutic dose of MTX with CA can be as effective as MTX in the therapeutic dose or even better.

## 2. Results

### 2.1. Change in Body Weight

**Figure 1:**
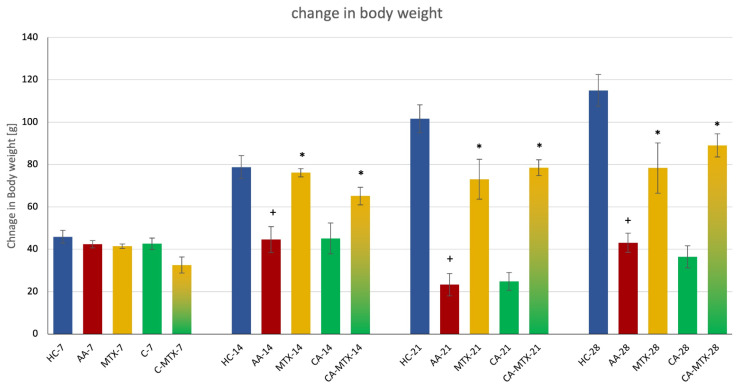
Change in body weight (day n (g) − day 1 (g) = increase in weight (g)) on the 7th, 14th, 21st, and 28th day. + HC vs. AA, * AA vs. MTX/CA/CA-MTX, *p* ≤ 0.05.

On the 7th day there were no significant changes in this parameter observable. On days 14, 21, and 28 there was a significant increase in body weight in the MTX and CA-MTX groups compared to the AA group (Figure 1) (*p* ≤ 0.05, AA vs. MTX/CA-MTX). Progressive weight gain of the HC Lewis animals was in the physiological range [32].

### 2.2. Hind Paw Volume

**Figure 2:**
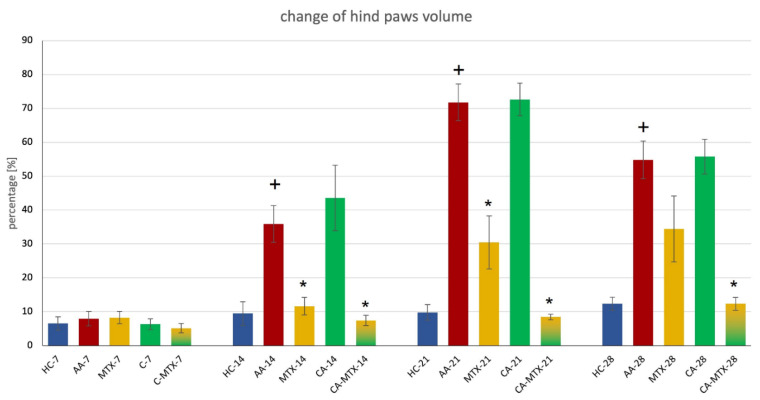
Change in the volume of the hind paws on the 7th, 14th, 21st, and 28th day. + HC vs. AA, * AA vs. MTX/CA/CA-MTX, *p* ≤ 0.05.

On the 14th, 21st, and 28th day there was a significant increment in hind paw volume in the AA group compared to the HC group (Figure 2) (*p* ≤ 0.05, HC vs. AA), which indicates an onset of arthritis, while on the 7th day there were no significant changes observable. On days 14 and 21 a significant decrease in hind paw volume was observed in the MTX and CA-MTX groups compared to the AA group (Figure 2) (*p* ≤ 0.05, AA vs. MTX/CA-MTX). On the 28th day there was a significant decrease in hind paw volume only in the CA-MTX group compared to the AA group (Figure 2) (*p* ≤ 0.05, AA vs. CA-MTX).

### 2.3. Plasmatic Interleukin-17A

**Figure 3:**
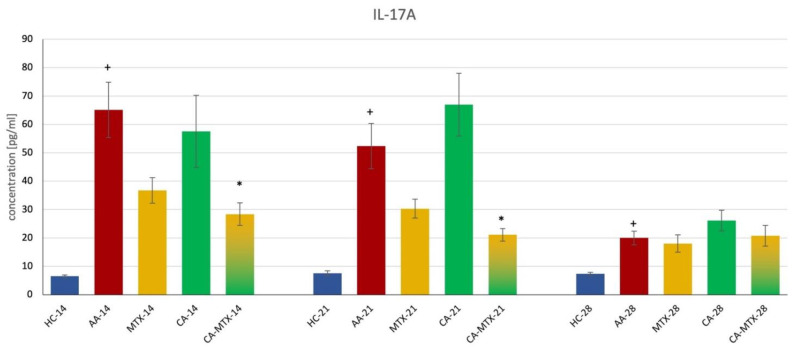
Interleukin 17A (IL-17A) levels in plasma on 14th, 21st, and 28th day. + HC vs. AA, * AA vs. MTX/CA/CA-MTX, *p* ≤ 0.05.

IL-17A was significantly increased in the untreated animal group (AA) compared to the healthy control group (HC) on all three monitored days (Figure 3) (*p* ≤ 0.05, HC vs. AA) indicating an onset of adjuvant arthritis, as previously shown for the change in hind paw volume. On the 14th and 21st days, there was a significant decrease in IL-17A concentration in the group with combination therapy compared to the AA group (Figure 3) (*p* ≤ 0.05, AA vs. CA-MTX).

### 2.4. Matrix Metalloproteinase-9 in Plasma

**Figure 4:**
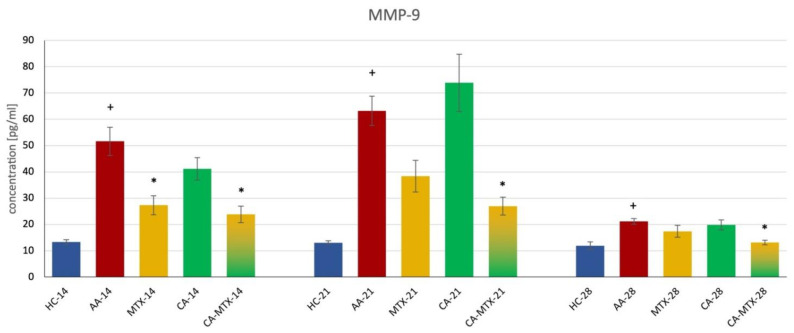
Levels of matrix metalloproteinase 9 (MMP-9) in plasma on the 14th, 21st, and 28th day. + HC vs. AA, * AA vs. MTX/CA/CA-MTX, *p* ≤ 0.05.

MMP-9 was significantly increased in the AA group during all three days (14th, 21st, 28th) compared to healthy animals (Figure 4) (*p* ≤ 0.05, HC vs. AA). On the 14th day, both MTX and CA-MTX groups had significantly lower levels of the MMP-9 in plasma compared to the AA group (Figure 4) (*p* ≤ 0.05, AA vs. MTX/CA-MTX). On the 21st and 28th days of the experiment, only the CA-MTX group significantly decreased the level of MMP-9 in plasma compared to the AA group (Figure 4) (*p* ≤ 0.05, AA vs. CA-MTX).

### 2.5. Monocyte Chemotactic Protein-1 in Plasma

**Figure 5:**
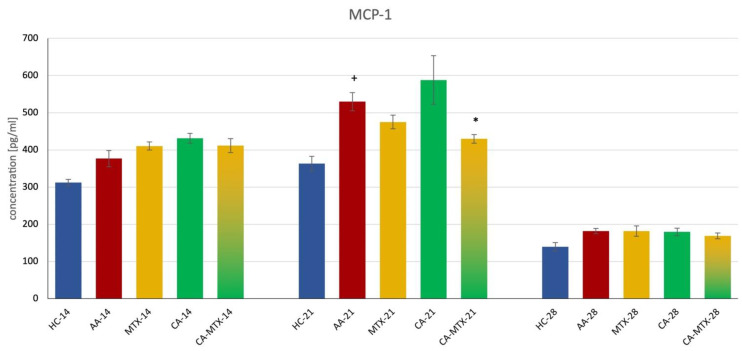
Monocyte chemotactic protein 1 (MCP-1) levels in plasma on the 14th, 21st, and 28th days. + HC vs. AA, * AA vs. MTX/CA/CA-MTX, *p* ≤ 0.05.

On the 14th and 28th days of the experiment there was no significant difference between the HC and AA group, as well as AA compared to the MTX and CA-MTX groups (Figure 5). On the 21st day, there was a significant increase in MCP-1 in the AA group compared to HC (Figure 5) (*p* ≤ 0.05, HC vs. AA). A significant decrease in MCP-1 was only seen in the CA-MTX group compared to AA (Figure 5) (*p* ≤ 0.05, AA vs. CA-MTX).

### 2.6. Activity of Gamma-Glutamyl Transferase in Joint

GGT was significantly increased in the AA group compared to the HC group (Table 1) (*p* ≤ 0.05, HC vs. AA). A significant decrease in GGT can be described in the CA-MTX group compared to the AA group (Table 1) (*p* ≤ 0.05, AA vs. CA-MTX).

### 2.7. Relative mRNA Expression of Interleukin-1β and Antioxidant Enzymes Heme Oxygenase (HO-1) and Catalase (CAT)

**Figure 6:**
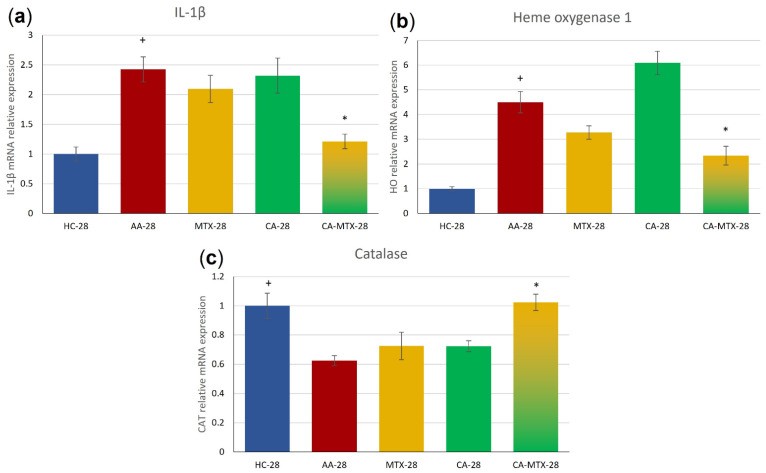
Relative mRNA expression of IL-1β (**a**), HO-1 (**b**) and CAT (**c**). + HC vs. AA, * AA vs. MTX/C/CA-MTX, *p* ≤ 0.05.

The gene expression of IL-1β was significantly increased in the AA group compared to HC (Figure 6a) (*p* ≤ 0.05, HC vs. AA). MTX itself did not exhibit an influence on this parameter (Figure 6a). IL-1β mRNA was significantly decreased only in the CA-MTX group compared to AA (Figure 6a) (*p* ≤ 0.05, AA vs. CA-MTX).

The gene expression of HO-1 was significantly increased in the AA group compared to the HC group (Figure 6b) (*p* ≤ 0.05, HC vs. AA). In CA monotherapy, a visible but nonsignificant increasing trend was observed compared to the AA group (Figure 6b). Methotrexate alone had no significant effect compared to the AA group (Figure 6b). HO-1 mRNA was significantly decreased in the CA-MTX group compared to the AA group (Figure 6b) (*p* ≤ 0.05, AA vs. CA-MTX). The catalase (CAT) mRNA level, as one of the antioxidant parameters, was significantly decreased in the AA group compared to HC (Figure 6c) (*p* ≤ 0.05, HC vs. AA). Again, the MTX group had no significant effect compared to the AA group (Figure 6c). A significant increase in CAT mRNA was observed in the CA-MTX group compared to AA (Figure 6c) (*p* ≤ 0.05, AA vs. CA-MTX).

## 3. Discussion

One of the most important aspects of our research is the combination treatment of a subtherapeutic dose of MTX (or other DMARDs) with natural compounds and extracts possessing anti-inflammatory, immunomodulatory, and antioxidant activities in AA resembling RA in patients. New combination therapies are therefore required to improve the available drug regimens to target the recent need [33]. Long-term treatment with NSAIDs, corticosteroids, and DMARDs, for the management of RA, results in harmful side effects. This calls for urgent new and safe options for the treatment of RA, using complementary medicine approaches. There is much evidence that has recommended supplementation with dietary, nutritional, and plant components, which could be important as adjuvants in reducing RA symptoms, through their effects on the excessive inflammatory processes. Dietary phenolic compounds, flavonoids, carotenoids, and alkaloids, with their ability to modulate pro-oxidant and pro-inflammatory pathways, have been effective in delaying the progression of arthritic disease. In addition, side effects associated with continuous treatment with antirheumatic drugs can be overcome using dietary, nutritional, and herbal interventions. This concept presents natural components that have the potential to promote health, improve general well-being, and reduce the risk of RA complications [34].

The primary and dominant processes in the etiopathogenesis of RA are immunological mechanisms, closely related to redox imbalance in the organism, which may enhance chronic inflammatory processes [35]. Furthermore, the production of ROS and reactive nitrogen species (RNS) by neutrophils and macrophages in inflamed tissue directly damages joint structure and functionality. Our studies agree with the findings of other authors who have referred to the important role of oxidative stress (OS) in the pathogenesis of RA [36,37,38]. In RA therapy, to date, combinations of classical immunosuppressive treatment with compounds possessing immunomodulatory and antioxidant activity have been used very rarely. For our experiments, we titrated the dose of MTX to 0.3 mg/kg twice a week. This dose inhibits the hind paw swelling by approximately 50% compared to the untreated AA group (Figure 2). This dosing allows the second experimental compound to show its effect if any. The therapeutic dose of MTX is usually 0.5–0.6 mg/kg twice a week [39,40].

Adjuvant arthritis in Lewis rats is a well-known and reliable model of RA. The main clinical manifestations of AA are paw swelling and the loss of body weight [41,42]. CA in monotherapy did not have a significant effect on hind paw swelling (Figure 2). In the literature we have found two animal studies on collagen-induced arthritis, where the authors demonstrate a significant reduction in hind paw swelling by the administration of CA [43,44]. This discrepancy from our result is probably due to the different arthritic models used and different doses administered to rats. However, the combination of CA + MTX was more effective in reducing hind paw volume than MTX in monotherapy (Figure 2). We are the first to report these results of MTX combination therapy with CA. 

Since MTX in monotherapy had a quite prominent protective effect on weight loss in AA animals, but the monotherapy with CA did not increase weight significantly, the combination of CA + MTX did not improve this effect on weight loss. Interestingly, Ibarra et al. evaluated the preventive effects of rosemary leaf extract that was standardized to 20% carnosic acid (RE) on weight gain, glucose levels, and lipid homeostasis in mice that had begun a high-fat diet (HFD) as juveniles. Body and epididymal fat weight in animals on the HFD that was supplemented with RE increased 69 and 79% less than those in the HFD group [45]. Similarly, Wang et al. [46] found that CA prevents obesity and hepatic steatosis in ob/ob mice. Our result, that CA in combination with MTX did not deteriorate the weight of AA animals, might be promising for patients with RA, who often suffer from arthritic cachexia [47].

In this experiment, we measured the levels of IL-17A, MMP-9, and MCP-1 in plasma. Chronic inflammation in AA significantly increased all these markers on days 14, 21, and 28 for IL-17A and MMP-9 (Figure 3 and Figure 4). The level of MCP-1 was significantly increased only on day 21 during this experiment (Figure 5). Interleukin-17A is a pro-inflammatory cytokine secreted by a subset of memory T cells and other innate immune cells. It is associated with RA due to IL-17A expression in RA synovial fluid. The severe bone erosive rat AA and mouse collagen-induced arthritis (CIA) models were used to address the therapeutic efficacy of anti-IL-17A treatment. In the AA model, treatment with anti-IL-17A completely alleviated arthritis, lowered the level of RANKL, and inhibited structural damage to the bones. In the CIA model, the neutralization of IL-17A coincident with arthritis development or in mice with established arthritis reduced joint swelling by inhibiting the onset and progression [48]. In the literature, there is information on the effect of CA on IL-17 in arthritic animal models. Liu et al. [44] showed a reduction in IL-17 expression in the fibroblast-like synoviocytes cell culture of RA patients by CA application. Xia et al. [43] found in a CIA model in db/db mice that CA administration reduced the serum level of IL-17. In our experiment, the monotherapy with CA was without effect on the level of IL-17 in AA rats. As mentioned above, our dissimilar results of CA monotherapy could be due to the different animal models and doses of CA (100 mg/kg in the current experiment) used. Xia et al. [43] administered the CA intraperitoneally in a dose of 5 mg/kg. Since CA was administered intraperitoneally, omitting liver metabolism, and at different doses, this could be the reason for the different result of CA in monotherapy. However, the combination treatment with CA + MTX was the most effective in decreasing IL-17A in AA. It seems that the strategy of the combination of two lower doses of effective substances can result in significant anti-inflammatory activity even if these two substances in monotherapy had no significant effects (see Figure 3). The results of a 30-day toxicity study indicated that a high dose of CA (600 mg/kg per day) could result in the injury of the liver and myocardial muscle, especially for male rats [49]. Therefore, it is possible to increase the dose of CA in male rats from 200 to 300 mg/kg a day and expect a better therapeutic profile in CA monotherapy.

In the present literature, we have not found results about the effect of CA on MMP-9 in rat animal models of RA. Our results show that CA only in combination with MTX can reduce the level of MMP-9 in plasma. Chae et al. [50] studied the effect of *Rosmarinus officinalis* L., CA, and carnosol on MMP-9 and MCP-1 levels, and cell migration in RAW 264.7 macrophages and smooth muscle cells. The activities of MMP-9 and MCP-1 decreased markedly with a methanolic rosemary extract, and CA in RAW 264.7 cells. The methanolic rosemary extract, CA, and carnosol suppressed TNF-α-induced vascular smooth muscle cell migration by inhibiting MMP-9 expression [45]. Very similar results were shown by Yu et al. [51]. CA inhibited MMP-9 activity and expression. Furthermore, ROS production and TNF-α-induced nuclear translocation of NF-κB p50 and p65 were dose-dependently suppressed by CA pretreatment. These results indicate that CA has anti-inflammatory properties and may prevent the migration of human aortic smooth muscle cells by suppressing the expression of MMP-9 through the down-regulation of NF-κB. We suppose that this mechanism could also be involved in reducing the level of MMP-9 in the plasma of AA rats by a combination of lower doses of CA and MTX. Additionally, MTX treatment of early-stage RA has been shown to decrease the levels of MMPs. Six months of treatment with MTX down-regulated serum concentrations of MMP-1, MMP-3, MMP-9, MMP-13, and TIMP-1 in patients with RA [52].

RA synovial tissue fibroblasts produce RANTES and MCP-1 after stimulation with TNF-α and IL-1β, mediators critical to the pathogenesis of RA [53]. Interestingly, in AA, MCP-1 is detected for the first time in synovial tissue and peripheral blood on day 18 after adjuvant injection when joint inflammation is already acute. These results suggest that the expression of RANTES precedes that of MCP-1 in AA, suggesting that RANTES may be more important at the initiation of disease and MCP-1 after disease onset [54]. In our experiment, only the combination of CA + MTX significantly reduced the level of MCP-1 in plasma on day 21 (see Figure 5). However, the administration of CA in a mice model of acetaminophen-induced liver toxicity decreased the expression of the mRNA of MCP-1 [55]. Wang et al. [56] demonstrated that CA protects against steatosis in ob/ob mice. CA reduced the expression of inflammatory cytokines such as IL-1β, IL-12, IL-17, IFN-γ, and MCP-1 in the liver [56]. Even if these results about the ability of CA to reduce MCP-1 expression in the liver are not from arthritic animal models, we hypothesize that CA could also act similarly in other tissues that are involved in arthritic inflammatory processes. The administration of MTX in CIA in rats resulted in a decreasing effect on the expression of the MCP-1 and TGF-β genes in joint tissue [57]. In a human study of paired synovial fluid mononuclear cells (SFMCs) from patients with RA, psoriatic arthritis, and spondylarthritis, MTX and other DMARDs were used and decreased MCP-1 production [58]. Furthermore, in one of our previous studies, MTX decreased the level of IL-1β and MCP-1 on day 14 in AA [59]. Therefore, we assume that the aforementioned mechanisms of CA and MTX may explain the effect of CA + MTX in subtherapeutic doses on the level of MCP-1 in plasma.

The activity of GGT is considered a reliable biochemical merit for inflammation and OS; its increased action in organs such as the spleen and joints is believed to be a good indicator of AA development at the systemic and local level [60].

There is no information about the activity of CA on GGT in joint tissue. We are the first to report that the combination of CA and MTX significantly reduced GGT activity in the joints of AA animals. In one of our previous studies with a combination of MTX and coenzyme Q_10_, we found out that the combination treatment was the most effective in reducing the activity of GGT in joints [61]. Furthermore, our team reported that GGT activity in tissues such as the spleen and joints could provide a simple and inexpensive marker for the development of AA and RA at both the systemic and the local level [62]. Since there are very few experiments aimed at studying the mechanism of MTX on GGT activity in arthritic diseases, it is still unclear how MTX affects the activity of GGT in joints.

The relative mRNA expression of IL-1β in the liver, an important organ of systemic inflammation, was increased in the untreated arthritic group in accordance with our previous results in the AA model [63]. Although monotherapies with MTX or CA did not exhibit an effect, the combination of both drugs showed a significant decrease in IL-1β mRNA, indicating an ability of CA to potentiate the anti-inflammatory effect of MTX. As MTX was shown to not be sufficiently effective in decreasing arthritis-stimulated IL-1β gene expression [64], the use of drugs such as CA capable of increasing the anti-inflammatory action of MTX could represent a promising supplement therapy.

The oxidative status (ROS formation, oxidation of lipids, proteins, and DNA) is elevated in patients with RA and animals with experimental arthritis [65,66]. CAT together with glutathione peroxidase and superoxide dismutase (SOD) are important members of the enzymatic antioxidant defense system. The activity of CAT was reported to be decreased under inflammatory conditions in RA patients [65]. In the liver of the AA model, an impaired ROS-scavenging system in arthritis was established by analyzing oxidative injury parameters, the levels and production of ROS, and antioxidative activities (diminished CAT activity) [66]. Our results show that the negative effect of inflammation on CAT activity is already at the level of gene transcription (AA group, Figure 6c). Interestingly, in the same study, superoxide dismutase (SOD) activity did not change in the arthritic liver in accordance with our results of the relative expression of SOD mRNA [66]. The combination of MTX and CA increased CAT mRNA to the level of a healthy control. There is a negative correlation of the CAT gene expression with the gene expression of pro-inflammatory cytokine IL-1β in the liver.

HO-1 is an antioxidant and anti-inflammatory enzyme induced in response to cytokines and ROS since its gene expression is under the control of pro-inflammatory transcription factors NF-κB and activator protein-1 (AP-1), and Nrf2, a sensor of oxidative and electrophilic stress [67,68,69]. At the protein level, immunoblotting assays were used to test whether CA affected HO-1 expression in articular chondrocytes. The results showed that CA increased enzyme levels in a dose-dependent manner. Furthermore, it was able to restore HO-1 levels under IL-1β treatment, which specifically inhibits the antioxidant effects of the enzyme. According to this study, the mechanisms by which this natural compound acts rely on the downregulation of MMP-13 and A disintegrin and metalloproteinase with thrombospondin motifs 5 (ADAMTS-5), on the activation of Nrf2, and on the regulation of the Kelch-like ECH-associated protein 1/Nrf2 (KEAP1/NRF2) transcriptional pathway [70]. The constitutive expression of HO-1 in chondrocytes and the meniscus in mice has been associated with preserving cartilage degeneration. For this reason, Hiroyuki et al. [71] explored the effect of CA as an inducer of the up-regulation of HO-1 in preventing the progression of osteoarthritis. As the gene expression of HO-1 is under the control of NF-κB, its increase in the AA group was expected. In our study, an observed marked, although nonsignificant, increase in HO-1 mRNA in the CA group could be the consequence of Nrf2 activation [68]. Considering IL-1β mRNA and other parameters, the decrease in HO-1 in the combination group (CA-MTX) could likely reflect the synergic anti-inflammatory effect of both drugs, potentially repressing NF-κB [68]. The decreased expression of the HO-1 gene and the increase in CAT mRNA in the CA-MTX group might indicate a decrease in oxidative stress.

## 4. Materials and Methods

### 4.1. Experimental Animals

The animals used for this experiment were Lewis bred male rats, which were obtained from the Department of Toxicology and Laboratory Animal Breeding, Centre of Experimental Medicine, SAS, Dobrá Voda, Slovakia (SK CH 24016). After the arrival of animals, they were housed in quarantine for seven days. Animals had access to tap water and food ad libitum, 12 h light/12 h dark cycle. The protocol of this experiment was approved by the Ethics Committee of the Institute of Experimental Pharmacology and Toxicology, Center of Experimental Medicine SAS in Bratislava, Slovakia (SK UCH 04018), and the State Veterinary and Food Administration of the Slovak Republic, Bratislava (3144/16-221/3) in accordance with the European Convention for the Protection of Vertebrate Animals Used for Experimental and Other Scientific Purposes.

### 4.2. Induction of Adjuvant Arthritis in Lewis Rats

Induction of AA was performed by single intradermal injection at the base of the tail. The solution used for induction (0.1 mL per animal) was an incomplete Freud’s adjuvant (Thermo Scientific, Rockford, IL, USA) in which heat-inactivated *Mycobacterium butyricum* (Becton, Dickinson and Company, Franklin Lakes, NJ, USA) was suspended. The weight of the animals used was between 160 g and 180 g. AA is a well-established model in which inflammation can manifest itself and can be easily identified [12].

### 4.3. Experimental Design and Treatment

The animals were divided into 5 groups, in each group 6–8 animals. The first group was healthy control animals without any treatment (HC group). The second group was animals with induced AA and without any treatment (AA group). The third group was animals with induced AA and with methotrexate treatment (Sandoz Pharmaceuticals d.d., Ljubljana, Slovenia) at a dose of 0.3 mg/kg twice a week (MTX group). The fourth group was animals with induced AA and carnosic acid treatment (Sigma-Aldrich, Saint-Louis, MO, USA) at a dose of 100 mg/kg daily (CA group). The fifth group was AA-induced animals with a combined treatment of methotrexate at a dose of 0.3 mg/kg twice a week and carnosic acid at a dose of 100 mg/kg daily (CA-MTX group). All substances were administered orally by gastric tube from the first day of induction. The experimental model was 28 days long. Blood samples were collected in tubes with heparin (Zentiva k.s., Prague, Czech Republic) and obtained from the retro-orbital plexus on the 14th and 21st days and punction from the heart on the 28th day under Zoletil (Virbac SA, Carros, France)/Xylariem (Ecuphar N.V., Oostkamp, Belgium) anesthesia. Measurements of body weight and paw volume were performed on the 7th, 14th, 21st, and 28th days. On the 28th day of the experiment, animals were sacrificed under deep anesthesia, and samples, blood, and tissue (liver, spleen, joint) were collected and put in a freezer at −80 °C. All parameters were measured from the 14th day due to the onset of the AA, which is usually around this day [72].

### 4.4. Change in Body Weight

Body weight (g) was measured before AA induction on the 1st day, and subsequently on the 7th, 14th, 21st, and 28th days. The change in body weight was determined as the difference in weight on the 7th, 14th, 21st, and 28th day and body weight that was measured on the 1st day. 

Formula: weight (g) = Day n − Day 1

n = day of measurement

### 4.5. Hind Paws’ Volume Change

The change in hind paws’ volume (%) is calculated as a percentage of increment of hind paws volume on the 7th, 14th, 21st, and 28th days compared to the measurements that were performed before the induction of AA (1st day) using a water plethysmometer (UGO BASILE, Comerio-Varese, Italy).

Formula: ([Day n]/[Day 1]) × 100 − 100 = value (%)

### 4.6. Markers of Inflammation

Interleukin 17A (IL-17A), matrix metalloproteinase 9 (MMP-9), and monocyte chemotactic protein-1 (MCP-1) were determined from plasma samples, which were collected on the 14th, 21st, and 28th days by using ELISA kits (Thermo Fisher Scientific, Waltham, MA, USA; R&D Systems Quantikine^®^, Minneapolis, MN, USA). Measurements were made as required in the instructions provided by the manufacturer available on their website.

### 4.7. The Activity of the Gamma-Glutamyltransferase in Joint

The cellular activity of gamma-glutamyltransferase (GGT) was measured on the 28th in the hind paw joint tissue homogenate using the method of Ondrejickova et al. [73]. The tissues were homogenized in a buffer (2.6 mM of NaH_2_PO_4_, 50 mM of Na_2_HPO_4_, 68 mM of NaCl, 15 mM of EDTA; pH 8.1) at 1:9 (*w*/*v*) by Ultra Turax TP 18/10 (IKA-Werke, Staufen, Germany) for one min at 0 °C. Biochemical substrates (44 mM of methionine and 8.7 mM of L-γ-glutamyl-p-nitroanilide) were dissolved in isopropyl alcohol (65%) to final concentrations of 2.5 mM and 12.6 mM, respectively. After an hour of incubation at 37 °C, the reaction was stopped by adding 2.3 mL of cold methanol. The tubes were centrifuged at 5000 rpm for 20 min (Centrifuge Eppendorf). The supernatant’s absorbance (product *p*-nitroaniline) was determined on the Specord 40 spectrophotometer (Analytik Jena, Jena, Germany) at λ = 406 nm. Solution mixes in the absence of either the substrate or acceptor were used as blanks. The activity was calculated on the basis of absorbance measurement using a calibration coefficient (*p*−nitroaniline).

### 4.8. RNA Extraction and Reverse Transcription

Total mRNA was isolated from liver tissue by RNAzol^®^ RT. Next 50–100 mg of liver samples were placed into 1.5 mL tubes with 600 μL of RNAzol and then homogenized. An amount of 240 μL of DEPC-treated sterile water was added to every sample. After intensive mixing of the samples, a 15 min incubation at room temperature followed. The samples were then centrifuged at 12,000× *g* for 15 min at 4 °C. Then 500 μL of supernatants were transferred to new 1.5 mL tubes, mixed with the same amount of 2-propanol, incubated on ice for 15 min, and then centrifugated at 12,000× *g* for 15 min at 4 °C. After centrifugation, 2-propanol was discarded, and the pellet was washed twice with 70% ethanol and centrifuged at 12,000× *g* for 10 min at 4 °C. When ethanol was discarded, the mRNA was solubilized in DEPC-treated water. The mRNA concentration was measured in a microplate reader (Epoch BioteK, Winooski, VT, USA) using absorption at λ = 260 nm and samples were checked for protein contamination by absorbance ratio 260/280. In total, 450 ng of isolated mRNA was then reverse transcribed into cDNA using the TaKaRa PrimeScript™ RT reagent kit according to the manufacturer’s protocol.

### 4.9. Quantitative Real-Time RT-PCR

After reverse transcription, cDNA was used for real-time polymerase chain reaction (quantitative RT-PCR) using HOT FIREPol EvaGreenR q PCR Mix Plus (Solis BioDyne, Tartu, Estonia) performed on QuantStudio™ 3 thermocycler (Applied Biosystems, Thermo Fisher Scientific, Foster City, CA, USA). The following oligonucleotide primers were used in the PCR reaction: β-actin forward: 5′-TCAAGATCATTGCTCCTCCTG-3′; reverse: 5′-AGGGTGTAAAACGCAGCTCA-3′; IL-1β forward: 5′-CCTCTGTGACTCGTGGGATG-3′; reverse: 5′-GGGTGTGCCGTCTTTCATCA-3′, HO-1 forward: 5′-TCACCTTCCCGAGCATCGAC-3′; reverse: 5′-GCAGCTCCTCAAACAGCTCAA-3′, CAT forward: 5′-GCACACTTTGACAGAGAGCG-3′; reverse: 5′-CTGACTCTCCAGCGACTGTG-3′. The thermal program consisted of initial activation at 95 °C for 15 min, followed by 40 cycles of 95 °C for 15 s, 60 °C for 30 s, and 72 °C for 30 s. The relative mRNA expression was analyzed by ΔΔCt method using β-actin as endogenous control. Primer specificity was verified by melt curve analysis and electrophoresis.

### 4.10. Statistical Analysis

Standard deviation, standard error of mean and mean values were calculated for each parameter in each group (six to eight animals in each experimental group). Statistically significant differences among treated, untreated, and control groups were tested using parametric Analysis of Variance (ANOVA). Post hoc tests (Welch’s (ANOVA)) were applied in situations where differences among groups were significant at the level of significance α = 0.05. After post hoc testing, the following significance levels were specified: significant (*p* < 0.05) and not significant (*p* > 0.05). The untreated arthritis group was compared with healthy control animals (+), and the treated arthritis groups was compared with untreated arthritic animals (*).

## 5. Conclusions

Combination therapies with methotrexate have been shown to be the most efficient treatment strategies for suppressing rheumatoid arthritis and stabilizing patients with low-level disease activity. In this experimental study with two lower doses of methotrexate and carnosic acid (CA + MTX) in combination, we have shown that, mainly, CA + MTX was the most therapeutically effective (reducing hind paw swelling, level of IL-17A, MMP-9, and MCP-1 in plasma and GGT in joint). Moreover, the mRNA expression of the markers of oxidative stress in the liver (HO-1 and CAT) and IL-1β were significantly improved only by CA + MTX. Our results indicate that adding carnosic acid to the methotrexate treatment could also be a viable therapeutic option for patients suffering from RA.

## Figures and Tables

**Table 1:** Levels of gamma-glutamyl transferase (GGT) in joint. + HC vs. AA, * AA vs. MTX/CA/CA-MTX, *p* ≤ 0.05.

Group	Mean
HC	11.46 ± 1.39
AA	20.94 ± 2.01 ^+^
MTX	12.48 ± 2.59
CA	17.88 ± 1.00
CA-MTX	10.95 ± 1.40 *

## Data Availability

The experimental data are reported here: https://figshare.com/articles/dataset/CA_data/21200257 (accessed on 23 September 2022).

## References

[1] Almutairi K.B., Nossent J.C., Preen D.B., Keen H.I., Inderjeeth C.A. (2021). The Prevalence of Rheumatoid Arthritis: A Systematic Review of Population-based Studies. J. Rheumatol..

[2] Mateen S., Zafar A., Moin S., Khan A.Q., Zubair S. (2016). Understanding the role of cytokines in the pathogenesis of rheumatoid arthritis. Clin. Chim. Acta.

[3] Rahman I., Bahgi D. (2014). Inflammation. Advancing Age and Nutrition.

[4] Van der Heide A., Jacobs J.W., Bijlsma J.W., Heurkens A.H., van Booma-Frankfort C., van der Veen M.J., Haanen H.C., Hofman D.M., van Albada-Kuipers G.A., ter Borg E.J. (1996). The effectiveness of early treatment with “second-line” antirheumatic drugs. A randomized, controlled trial. Ann. Intern. Med..

[5] Chetan G., Shinde M.P., Venkatesh T.M., Kumar P., Shivakumar H.G. (2014). Methotrexate: A Gold Standard for Treatment of Rheumatoid Arthritis. J. Pain Palliat. Care Pharmacother..

[6] Salliot C., van der Heijde D. (2009). Long-term safety of methotrexate monotherapy in patients with rheumatoid arthritis: A systematic literature research. Ann. Rheum. Dis..

[7] Cronstein B.N., Aune T.M. (2020). Methotrexate and its mechanisms of action in inflammatory arthritis. Nat. Rev. Rheumatol..

[8] Smolen J.S., Landewé R., Breedveld F.C., Dougados M., Emery P., Gaujoux-Viala C., Gorter S., Knevel R., Nam J., Schoels M. (2010). EULAR recommendations for the management of rheumatoid arthritis with synthetic and biological disease-modifying antirheumatic drugs. Ann. Rheum. Dis..

[9] Rubbert-Roth A., Finckh A. (2009). Treatment options in patients with rheumatoid arthritis failing initial TNF inhibitor therapy: A critical review. Arthritis Res. Ther..

[10] Pope J., Sawant R., Tundia N., Du E.X., Qi C.Z., Song Y., Tang P., Betts K.A. (2020). Comparative Efficacy of JAK Inhibitors for Moderate-To-Severe Rheumatoid Arthritis: A Network Meta-Analysis. Adv. Ther..

[11] Smolen J. (2018). Response to: “Concerns on Glucocorticoid Use for Japanese Patients with Established Rheumatoid Arthritis” by Oiwa. Ann. Rheum. Dis..

[12] Choudhary N., Bhatt L.K., Prabhavalkar K.S. (2018). Experimental animal models for rheumatoid arthritis. Immunopharmacol. Immunotoxicol..

[13] Roy T., Ghosh S. (2013). Animal models of rheumatoid arthritis: Correlation and usefulness with human rheumatoid arthritis. Indo Amer. J. Pharm. Res..

[14] Stolina M., Bolon B., Middleton S., Dwyer D., Brown H., Duryea D., Zhu L., Rohner A., Pretorius J., Kostenuik P. (2009). The evolving systemic and local biomarker milieu at different stages of disease progression in rat adjuvant-induced arthritis. J. Clin. Immunol..

[15] Kim I.S., Yang W.S., Kim C.H. (2022). Physiological Properties, Functions, and Trends in the Matrix Metalloproteinase Inhibitors in Inflammation-Mediated Human Diseases. Curr. Med. Chem..

[16] Firestein G.S. (2003). Evolving concepts of rheumatoid arthritis. Nature.

[17] Ayala J.M., Goyal S., Liverton N.J., Claremon D.A., O’Keefe S.J., Hanlon W.A. (2000). Serum-induced monocyte differentiation and monocyte chemotaxis are regulated by the p38 MAP kinase signal transduction pathway. J. Leukoc. Biol..

[18] Boring L., Gosling J., Chensue S.W., Kunkel S.L., Farese R.V., Broxmeyer H.E., Charo I.F. (1997). Impaired monocyte migration and reduced type 1 (Th1) cytokine responses in C-C chemokine receptor 2 knockout mice. J. Clin. Investig..

[19] Volin M.V., Shah M.R., Tokuhira M., Haines G.K., Woods J.M., Koch A.E. (1998). RANTES expression and contribution to monocyte chemotaxis in arthritis. Clin. Immunol. Immunopathol..

[20] Ulrich-Merzenich G.S. (2014). Combination screening of synthetic drugs and plant derived natural products—Potential and challenges for drug development. Synergy.

[21] Birtić S., Dussort P., Pierre F.-X., Bily A.C., Roller M. (2015). Carnosic acid. Phytochemistry.

[22] Zheng H., Li J., Ning F., Wijaya W., Chen Y., Xiao J., Huang Q. (2021). Improving in vitro bioaccessibility and bioactivity of carnosic acid using a lecithin-based nanoemulsion system. Food Funct..

[23] Zhang Y., Smuts J.P., Dodbiba E., Rangarajan R., Lang J.C., Armstrong D.W. (2012). Degradation study of carnosic acid, carnosol, rosmarinic acid, and rosemary extract (*Rosmarinus officinalis* L.) assessed using HPLC. J. Agric. Food Chem..

[24] Koutsoulas A., Čarnecká M., Slanina J., Tóth J., Slaninová I. (2019). Characterization of Phenolic Compounds and Antiproliferative Effects of Salvia pomifera and Salvia fruticosa Extracts. Molecules.

[25] Abadi M.N.A., Mortazavi M., Kalani N., Marzouni H.Z., Kooti W., Ali-Akbari S. (2016). Effect of hydroalcoholic extract of *Rosmarinus officinalis* L. leaf on anxiety in mice. Evid. Based Complement. Altern. Med..

[26] Zhao Y., Sedighi R., Wang P., Chen H., Zhu Y., Sang S. (2015). Carnosic acid as a major bioactive component in rosemary extract ameliorates high-fat-diet-induced obesity and metabolic syndrome in mice. J. Agric. Food Chem..

[27] Erkan N., Ayranci G., Ayranci E. (2008). Antioxidant activities of rosemary (*Rosmarinus Officinalis* L.) extract, blackseed (*Nigella sativa* L.) essential oil, carnosic acid, rosmarinic acid and sesamol. Food Chem..

[28] Yanagitai M., Itoh S., Kitagawa T., Takenouchi T., Kitani H., Satoh T. (2012). Carnosic acid, a pro-electrophilic compound, inhibits LPS-induced activation of microglia. Biochem. Biophys. Res. Commun..

[29] Li X., Zhao L., Han J.J., Zhang F., Liu S., Zhu L., Wang Z.Z., Zhang G.X., Zhang Y. (2018). Carnosol Modulates Th17 Cell Differentiation and Microglial Switch in Experimental Autoimmune Encephalomyelitis. Front Immunol..

[30] Khella K.F., el Maksoud A.I.A., Hassan A., Abdel-Ghany S.E., Elsanhoty R.M., Aladhadh M.A., Abdel-Hakeem M.A. (2022). Carnosic Acid Encapsulated in Albumin Nanoparticles Induces Apoptosis in Breast and Colorectal Cancer Cells. Molecules.

[31] Den Hartogh D.J., Vlavcheski F., Giacca A., MacPherson R.E.K., Tsiani E. (2022). Carnosic Acid Attenuates the Free Fatty Acid-Induced Insulin Resistance in Muscle Cells and Adipocytes. Cells.

[32] Growth Chart. https://www.criver.com/products-services/find-model/lewis-rat?region=3616#panel1-growth-chart.

[33] Burmester G., Feist E., Dörner T. (2014). Emerging cell and cytokine targets in rheumatoid arthritis. Nat. Rev. Rheumatol..

[34] Sharma D., Chaubey P., Suvarna V. (2021). Role of natural products in alleviation of rheumatoid arthritis—A review. J. Food Biochem..

[35] Kunsch C., Sikorski J.A., Sundell C.L. (2005). Oxidative stress and the use of antioxidants for the treatment of rheumatoid arthritis. Curr. Med. Chem. Immunol. Endoc. Metab. Agents.

[36] Bauerová K., Bezek A. (1999). Role of reactive oxygen and nitrogen species in etiopathogenesis of rheumatoid arthritis. Gen. Physiol. Biophys..

[37] Kundu S., Ghosh P., Datta S., Ghosh A., Chattopadhyay S., Chatterjee M. (2012). Oxidative stress as a potential biomarker for determining disease activity in patients with rheumatoid arthritis. Free Radic. Res..

[38] Stamp L.K., Khalilova I., Tarr J.M., Senthilmohan R., Turner R., Haigh R.C., Winyard P.G., Kettle A.J. (2012). Myeloperoxidase and oxidative stress in rheumatoid arthritis. Rheumatology.

[39] Rovenský J., Stancíková M., Svík K., Utesený J., Bauerová K., Jurcovicová J. (2009). Treatment of adjuvant-induced arthritis with the combination of methotrexate and probiotic bacteria Escherichia coli O83 (Colinfant). Folia Microbiol..

[40] Refaat R., Salama M., Meguid E.A., el Sarha A., Gowayed M. (2013). Evaluation of the effect of losartan and methotrexate combined therapy in adjuvant-induced arthritis in rats. Eur. J. Pharmacol..

[41] Van Eden W., Wage-naar-Hilbers J.P., Wauben M.H. (2001). Adjuvant arthritis in the rat. Curr. Protoc. Immunol..

[42] Tsiklauri L., Švík K., Chrastina M., Poništ S., Dráfi F., Slovák L., Alania M., Kemertelidze E., Bauerova K. (2021). Bio-flavonoid Robinin from Astragalus falcatus Lam. Mildly Improves the Effect of Metothrexate in Rats with Adjuvant Arthritis. Nutrients.

[43] Xia G., Wang X., Sun H., Qin Y., Fu M. (2017). Carnosic acid (CA) attenuates collagen-induced arthritis in db/db mice via inflammation suppression by regulating ROS-dependent p38 pathway. Free Radic. Biol. Med..

[44] Liu M., Zhou X., Zhou L., Liu Z., Yuan J., Cheng J., Zhao J., Wu L., Li H., Qiu H. (2018). Carnosic acid inhibits inflammation response and joint destruction on osteoclasts, fibroblast-like synoviocytes, and collagen-induced arthritis rats. J. Cell. Physiol..

[45] Ibarra A., Cases J., Roller M., Chiralt-Boix A., Coussaert A., Ripoll C. (2011). Carnosic acid-rich rosemary (*Rosmarinus officinalis* L.) leaf extract limits weight gain and improves cholesterol levels and glycaemia in mice on a high-fat diet. Br. J. Nutr..

[46] Wang T., Takikawa Y., Satoh T., Yoshioka Y., Kosaka K., Tatemichi Y., Suzuki K. (2011). Carnosic acid prevents obesity and hepatic steatosis in ob/ob mice. Hepatol. Res..

[47] Santo R.C.E., Fernandes K.Z., Lora P.S., Filippin L.I., Xavier R.M. (2018). Prevalence of rheumatoid cachexia in rheumatoid arthritis: A systematic review and meta-analysis. J. Cachexia Sarcopenia Muscle.

[48] Chao C.C., Chen S.J., Adamopoulos I.E., Davis N., Hong K., Vu A., Kwan S., Fayadat-Dilman L., Asio A., Bowman E.P. (2011). Anti-IL-17A therapy protects against bone erosion in experimental models of rheumatoid arthritis. Autoimmunity.

[49] Wang Q.L., Li H., Li X.X., Cui C.Y., Wang R., Yu N.X., Chen L.X. (2012). Acute and 30-day oral toxicity studies of administered carnosic acid. Food Chem. Toxicol..

[50] Chae I.G., Yu M.H., Im N.K., Jung Y.T., Lee J., Chun K.S., Lee I.S. (2012). Effect of Rosemarinus Officinalis L. on MMP-9, MCP-1 levels, and cell migration in RAW 264.7 and smooth muscle cells. J. Med. Food.

[51] Yu Y.M., Lin H.C., Chang W.C. (2008). Carnosic acid prevents the migration of human aortic smooth muscle cells by inhibiting the activation and expression of matrix metalloproteinase-9. Br. J. Nutr..

[52] Fiedorczyk M., Klimiuk P.A., Sierakowski S., Gindzienska-Sieskiewicz E., Chwiecko J. (2006). Serum matrix metalloproteinases and tissue inhibitors of metalloproteinases in patients with early rheumatoid arthritis. J. Rheumatol..

[53] Hosaka S., Akahoshi T., Wada C., Kondo H. (1994). Expression of the chemokine superfamily in rheumatoid arthritis. Clin. Exp. Immunol..

[54] Shahrara S., Proudfoot A.E., Park C.C., Volin M.V., Haines G.K., Woods J.M., Aikens C.H., Handel T.M., Pope R.M. (2008). Inhibition of monocyte chemoattractant protein-1 ameliorates rat adjuvant-induced arthritis. J. Immunol..

[55] Guo Q., Shen Z., Yu H., Lu G., Yu Y., Liu X., Zheng P. (2016). Carnosic acid protects against acetaminophen-induced hepatotoxicity by potentiating Nrf2-mediated antioxidant capacity in mice. Korean J. Physiol. Pharmacol..

[56] Wang T., Takikawa Y., Tabuchi T., Satoh T., Kosaka K., Suzuki K. (2012). Carnosic acid (CA) prevents lipid accumulation in hepatocytes through the EGFR/MAPK pathway. J. Gastroenterol..

[57] Jabbari N., Eftekhari Z., Roodbari N.H., Parivar K. (2020). Evaluation of Encapsulated Eugenol by Chitosan Nanoparticles on the aggressive model of rheumatoid arthritis. Int. Immunopharmacol..

[58] Nielsen M.A., Lomholt S., Mellemkjaer A., Andersen M.N., Buckley C.D., Kragstrup T.W. (2020). Responses to Cytokine Inhibitors Associated with Cellular Composition in Models of Immune-Mediated Inflammatory Arthritis. ACR Open Rheumatol..

[59] Kuncirova V., Ponist S., Mihalova D., Drafi F., Nosal R., Acquaviva A., Gardi C., Harmatha J., Hradkova I., Bauerova K. (2014). N-feruloylserotonin in preventive combination therapy with methotrexate reduced inflammation in adjuvant arthritis. Fundam. Clin. Pharmacol..

[60] Tsiklauri L., Drafi F., Poništ S., Slovák L., Chrastina M., Švík K., Kemoklidze Z., Kemertelidze E., Bauerová K. (2019). Study of anti-inflammatory activity of Fatsiphloginum™ (Fatsia japonica) and a new purified triterpene-rich extract of saponins (PS-551) in an experimental model of arthritis. Physiol. Res..

[61] Bauerova K., Paulovicova E., Mihalova D., Drafi F., Strosova M., Mascia C., Biasi F., Rovensky J., Kucharska J., Gvozdjakova A. (2010). Combined methotrexate and coenzyme Q₁₀ therapy in adjuvant-induced arthritis evaluated using parameters of inflammation and oxidative stress. Acta Biochim. Pol..

[62] Bauerová K., Ponist S., Ondrejicková O., Komendová D., Mihalová D. (2006). Association between tissue gamma-glutamyl-transferase and clinical markers of adjuvant arthritis in Lewis rats. Neuro Endocrinol. Lett..

[63] Pašková Ľ., Kuncírová V., Poništ S., Mihálová D., Nosáľ R., Harmatha J., Hrádková I., Čavojský T., Bilka F., Šišková K. (2016). Effect of N-Feruloylserotonin and Methotrexate on Severity of Experimental Arthritis and on Messenger RNA Expression of Key Proinflammatory Markers in Liver. J. Immunol. Res..

[64] Häupl T., Yahyawi M., Lübke C., Ringe J., Rohrlach T., Burmester G.R., Sittinger M., Kaps C. (2007). Gene expression profiling of rheumatoid arthritis synovial cells treated with antirheumatic drugs. J. Biomol. Screen.

[65] Mateen S., Moin S., Khan A.Q., Zafar A., Fatima N. (2016). Increased Reactive Oxygen Species Formation and Oxidative Stress in Rheumatoid Arthritis. PLoS ONE.

[66] Comar J.F., de Sá-Nakanishi A.B., de Oliveira A.L., Wendt M.M.N., Amado C.A.B., Iwamoto E.L.I., Peralta R.M., Bracht A. (2013). Oxidative state of the liver of rats with adjuvant-induced arthritis. Free Radic. Biol. Med..

[67] Yamamoto M., Kensler T.W., Motohashi H. (2018). The KEAP1-NRF2 System: A Thiol-Based Sensor-Effector Apparatus for Maintaining Redox Homeostasis. Physiol. Rev..

[68] Funes S.C., Rios M., Fernández-Fierro A., Covián C., Bueno S.M., Riedel C.A., Mackern-Oberti J.P., Kalergis A.M. (2020). Naturally Derived Heme-Oxygenase 1 Inducers and Their Therapeutic Application to Immune-Mediated Diseases. Front. Immunol..

[69] Alam J., Cook J.L. (2007). How many transcription factors does it take to turn on the heme oxygenase-1 gene?. Am. J. Respir. Cell Mol. Biol..

[70] Bahri S., Jameleddine S., Shlyonsky V. (2016). Relevance of carnosic acid to the treatment of several health disorders: Molecular targets and mechanisms. Biomed. Pharmacother..

[71] Ishitobi H., Sanada Y., Kato Y., Ikuta Y., Shibata S., Yamasaki S., Lotz M.K., Matsubara K., Miyaki S., Adachi N. (2018). Carnosic acid attenuates cartilage degeneration through induction of heme oxygenase-1 in human articular chondrocytes. Eur. J. Pharmacol..

[72] Bina J., Wilder R.L. (1999). Animal models of rheumatoid arthritis. Mol. Med. Today.

[73] Ondrejickova O., Ziegelhoeffer A., Gabauer I., Sotnikova R., Styk J., Gibala P., Sedlak J., Horakova L. (1993). Evaluation of ischemia-reperfusion injury by malondialdehyde, glutathione and gamma-glutamyltranspeptidase: Lack of specific localeffects in diverse parts of the dog heart following acute coronary occlusion. Cardioscience.

